# Distinct cell types in the superficial superior colliculus project to the dorsal lateral geniculate and lateral posterior thalamic nuclei

**DOI:** 10.1152/jn.00248.2018

**Published:** 2018-06-13

**Authors:** Samuel D. Gale, Gabe J. Murphy

**Affiliations:** Janelia Research Campus, Ashburn, Virginia

**Keywords:** LGN, mouse, pulvinar, superior colliculus, thalamus

## Abstract

The superficial layers of the superior colliculus (sSC) receive retinal input and project to thalamic regions, the dorsal lateral geniculate (dLGN) and lateral posterior (LP; or pulvinar) nuclei, that convey visual information to cortex. A critical step toward understanding the functional impact of sSC neurons on these parallel thalamo-cortical pathways is determining whether different classes of sSC neurons, which are known to respond to different features of visual stimuli, innervate overlapping or distinct thalamic targets. Here, we identified a transgenic mouse line that labels sSC neurons that project to dLGN but not LP. We utilized selective expression of fluorophores and channelrhodopsin in this and previously characterized mouse lines to demonstrate that distinct cell types give rise to sSC projections to dLGN and LP. We further show that the glutamatergic sSC cell type that projects to dLGN also provides input to the sSC cell type that projects to LP. These results clarify the cellular origin of parallel sSC-thalamo-cortical pathways and reveal an interaction between these pathways via local connections within the sSC.

**NEW & NOTEWORTHY** The superficial layers of the superior colliculus (sSC) project to two visual thalamic targets: the dorsal lateral geniculate (dLGN) and lateral posterior (LP) nuclei. We show that distinct excitatory sSC cell types give rise to these projections; stellate cells project to dLGN and wide-field (WF) cells project to LP. Moreover, these pathways interact via a connection within the sSC from stellate to WF cells.

## INTRODUCTION

The superficial layers of the superior colliculus (sSC) receive input from nearly every retinal ganglion cell and visual cortical area (Ellis et al. 2016; Wang and Burkhalter 2013), suggesting an important role in visually guided behavior. The sSC projects to multiple downstream areas including the dorsal lateral geniculate (dLGN) and lateral posterior (LP; or pulvinar) thalamic nuclei. These regions project to and receive input from primary visual cortex (V1) and, in the case of LP, higher order visual cortical areas (May 2006; Oh et al. 2014; Roth et al. 2016; Zhou et al. 2018). These connections likely endow the sSC with important roles in shaping the propagation of visual information to and between visual cortical areas.

Distinct sSC cell types with different visual response properties are thought to mediate projections to dLGN and LP (Gale and Murphy 2014; May 2006). Retrograde tracer injections in LP primarily label neurons with somas in the deepest portion of the sSC (the optic fiber layer) and dendritic morphology characteristic of wide-field (WF) cells (Zhou et al. 2017). Injections in dLGN primarily label cells in the upper layers of the sSC, which are presumed to be narrow-field (NF) and/or stellate cells (Albano et al. 1979; Graham and Casagrande 1980; Harting et al. 1991; Sugita et al. 1983). A study using antidromic stimulation of sSC neurons yielded roughly similar results: the majority of neurons antidromically activated by LP stimulation were WF cells (although some were identified as NF or stellate cells), and most dLGN-projecting neurons were described as NF, stellate, or marginal cells (Mooney et al. 1988).

These and other results describing sSC connectivity are limited by two technical constraints. First, identification of sSC cell types is typically based on qualitative features of somatodendritic morphology rather than objective methods. Second, dLGN and LP lie in close proximity and use of retrograde tracers or antidromic stimulation to identify particular thalamic targets of sSC neurons is potentially confounded by fibers of passage.

To address these challenges, we recently used quantitative morphological and electrophysiological properties and transgenic mouse lines expressing Cre-recombinase in subsets of cells to define four sSC cell types comparable to those previously described (Gale and Murphy 2014). We utilized Cre-dependent fluorophore expression and retrograde labeling to identify the axonal targets of each sSC cell type. Consistent with other studies, we found that WF cells project to LP but not dLGN and that a small subset of horizontal cells, which are GABAergic, project to dLGN (Bickford et al. 2015; Endo et al. 2003; Gale and Murphy 2014). Both anterograde and retrograde tracing failed to reveal a NF cell projection to dLGN or LP. Stellate cells, therefore, are the most likely source of sSC glutamatergic input to dLGN (Bickford et al. 2015).

Although retrograde labeling confirmed a stellate cell to thalamus projection (Gale and Murphy 2014), the fibers of passage problem and lack of a Cre line that specifically labels stellate cells prevented us from confirming that stellate cells project to dLGN or whether any stellate cells project to LP. Here, taking advantage of a more recently generated transgenic line in which stellate but not WF cells express Cre, we sought to clarify the cellular origin of sSC-thalamic pathways and the interconnectivity of sSC cell types by evaluating: *1*) which sSC cells provide glutamatergic input to dLGN; *2*) do any of these cells also project to LP; and *3*) do cells projecting to dLGN or LP interact via local connections within sSC?

## METHODS

### 

#### Transgenic mice and virus injections.

All procedures were approved by the Janelia Research Campus Institutional Animal Care and Use Committee and the Allen Institute for Brain Science. C57BL/6J mice of either sex were 6–18 wk old at the time of experiments. For some experiments, we used transgenic mice expressing Cre recombinase in subsets of cells: Gad2 Cre, Grp-KH288 Cre, Ntsr1-GN209 Cre, or Rorb Cre (Gerfen et al. 2013; Harris et al. 2014; Taniguchi et al. 2011). Note that the Ntsr1-GN209 Cre line differs substantially from the more commonly used Ntsr1-GN220 Cre line (Gerfen et al. 2013). To enable Cre-dependent fluorophore expression, we crossed the Cre lines listed above to Ai9, Ai14, or Ai32 mice (Madisen et al. 2012) or injected AAV-2.1-Flex-Syn-green fluorescent protein (GFP) into the sSC. For experiments utilizing Cre-dependent channelrhodopsin (ChR2) expression, we injected AAV-2.1-FLEX-Syn-ChR2-GFP in the sSC. Two 20-nl virus injections were made at the following coordinates (in millimeters anterior from lambda, lateral from midline, and ventral from pia): 0.3, 0.3, 1.0 and 0.1, 0.8, 1.0. In vitro electrophysiological recordings were performed 4–5 wk after virus injection. We did not observe GFP-labeled cell bodies in any region outside of sSC. Regions reciprocally connected with the sSC [ventral LGN (vLGN) and parabigeminal nucleus (PBG)] were not present in slices from which sSC neurons were recorded.

#### In vitro electrophysiology.

Parasagittal brain slices (400-μm thick) were cut with a vibratome (Leica) in chilled cutting solution containing the following (in mM): 60 sucrose, 83 NaCl, 25 NaHCO_3_, 1.25 NaH_2_PO_4_, 2.5 KCl, 0.5 CaCl_2_, 6 MgCl_2_, 20 d-glucose, 3 Na pyruvate, and 1 ascorbic acid. Slices were transferred to warm (34°C) cutting solution, which was then allowed to cool to room temperature. Approximately 60 min after cutting, slices were transferred to artificial cerebral spinal fluid containing the following (in mM): 125 NaCl, 25 NaHCO_3_, 1.25 NaH_2_PO_4_, 2.5 KCl, 1.3 CaCl_2_, 1 MgCl_2_, 20 d-glucose, 3 Na pyruvate, and 1 ascorbic acid for recording (at 32°C) or further storage (room temperature). For some recordings, the AMPA receptor antagonist NBQX (10 μM), the NMDA receptor antagonist AP5 (50 μM), or the GABA-A receptor antagonist gabazine (10 μM) was added to the artificial cerebral spinal fluid perfusing the slice.

Whole cell, current-clamp recordings were made with glass pipettes filled with the following (in mM): 134 K gluconate, 6 KCl, 4 NaCl, 10 HEPES, 2 MgATP, 0.4 NaGTP, 10 Tris phosphocreatine, and 0.05 Na Alexa Fluor 488 or 594 hydrazide. Electrode resistance was 3–8 MΩ. Membrane voltage was amplified 50× and low-pass filtered (4 kHz cutoff) with a Multiclamp 700B amplifier (Molecular Devices) and digitized at 50 kHz with an ITC-18 data acquisition interface (Heka). ChR2 was activated with 1–1,000 ms LED flashes (470 nm peak emission) delivered through a ×63 objective. At the end of each recording, before the electrode was pulled off the cell, Alexa Fluor filling the cell and Cre-dependent fluorophore expression were imaged with a Bruker 2-photon microscope. Cre-dependent fluorophore expression was not used to target specific cells for recordings.

#### Data analysis.

sSC cell types were identified in vitro via their distinct electrophysiological properties as described previously (Gale and Murphy 2014). In brief, 11 electrophysiological parameters were calculated for each cell and hierarchical clustering into 4 groups was performed using Ward’s linkage criterion. Previously, we also clustered sSC cells using somato-dendritic morphological properties (Gale and Murphy 2014). The four clusters based on electrophysiological properties alone were nearly identical in membership to four clusters based solely on morphological properties of the same cells. We chose existing nomenclature based on qualitative morphological properties to label the cell types defined by each cluster (WF, NF, stellate, and horizontal). Merging or splitting these clusters into more or less than four cell types resulted in inconsistencies between clusters defined by electrophysiological or morphological properties. For the present study, we chose to use electrophysiological properties for cell type identification because these parameters are easily acquired for each cell, whereas quantification of morphological properties requires an extended time to fill (with Alexa Fluor) and image the full dendritic arbor (in particular for WF cells).

For quantifying the amplitude of synaptic responses (see Figs. 2*C* and 3*C*), membrane voltage (*V*_m_) traces were median filtered (10-ms window) to reduce the impact of action potentials when present. The “peak response” to brief (<10 ms) light pulses was calculated as the average difference between the peak *V*_m_ (maximum or minimum) during the first 100 ms after stimulus onset and the last 100 ms before stimulus onset. The “sustained response” to 1000 ms light pulses was calculated as the difference between the average *V*_m_ from 500 to 1,000 ms after stimulus onset and the average *V*_m_ before stimulus onset. To determine if a significant response was observed in a given cell, we compared peak *V*_m_ before and after stimulus offset (minimum 5 trials) using two-sided Wilcoxon signed-rank tests with α = 0.05.

## RESULTS

Cre-expressing cells in Rorb Cre transgenic mice (Harris et al. 2014) are dense in the upper sSC layers but not the optic fiber layer, where the somas of most WF cells reside. This led us to believe that the Rorb Cre line might facilitate labeling and manipulation of stellate cells independently of WF cells.

**Fig. 1.**
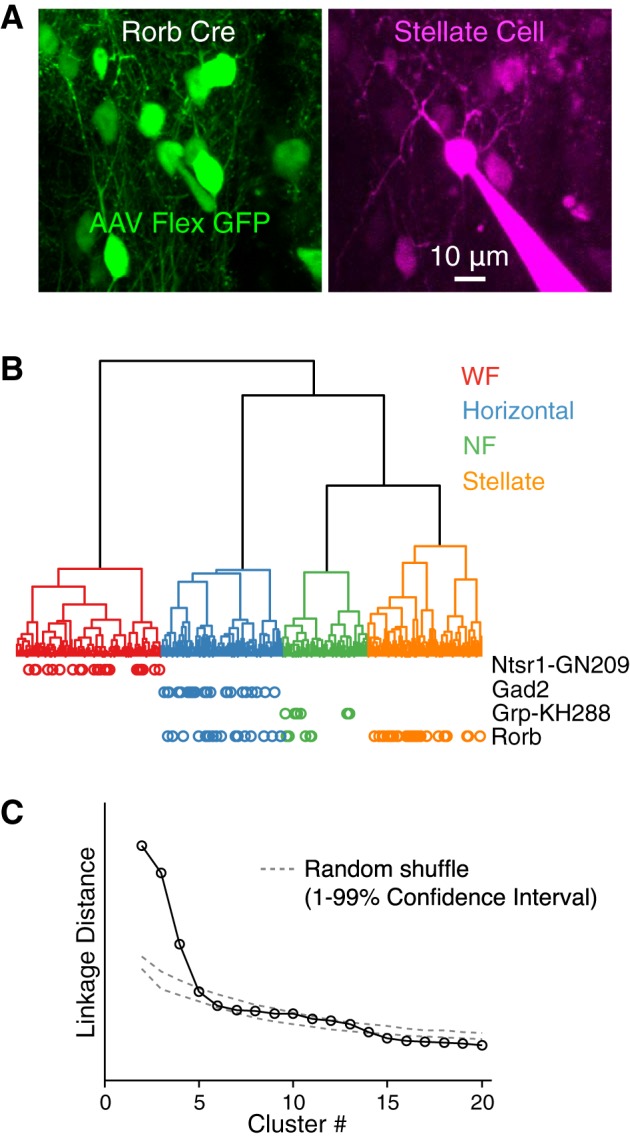
Nearly all stellate cells but not wide-field (WF) cells express Cre-dependent reporter in Rorb Cre mice. *A*: example green fluorescent protein (GFP)-expressing stellate cell recorded in superficial layers of the superior colliculus (sSC) in vitro after injection of AAV-Flex-ChR2-GFP in a Rorb Cre mouse. Note that some bright GFP-expressing cells are visible in the magenta channel, but the Alexa Flour 594 filling the recorded cell is not visible in the green channel. *B*: dendrogram representing, via vertical line lengths, the separation (linkage distance) of cells based on electrophysiological properties recorded in vitro. Vertical line ends at the bottom on the dendrogram represent individual cells (*n* = 878). Horizontal lines join similar cells into clusters. The 4 clusters shown in different colors correspond to the 4 cell types described by Gale and Murphy 2014. A subset of cells (*n* = 317) were recorded in 1 of 4 Cre lines (Table 1); cells from these experiments that expressed Cre-dependent reporter are indicated by colored circles below each cell’s position in the dendrogram. *C*: linkage distance between the 20 largest clusters in the dendrogram shown in *A*. Linkage distance is the increase in total within cluster variance (sum of squares of the Euclidean distance between each cell within a cluster and the cluster centroid in electrophysiological parameter space) that results from merging cluster N with cluster N-1. Dashed gray lines show the 1–99% confidence interval of linkage distances for clusters formed from randomly shuffled data. To generate randomly shuffled cluster data (1,000 repetitions), for each electrophysiological parameter, each cell was assigned a value for that parameter that was randomly drawn from the values of that parameter across all cells.

To identify which sSC cells express Cre in Rorb Cre mice, we performed whole cell patch-clamp recordings in brain slices of Rorb Cre mice that had been crossed with the Ai14 reporter line or injected in sSC with virus enabling Cre-dependent fluorophore expression (Fig. 1*A*). Combined with previous data (including similar experiments with other Cre lines; Table 1), we divided cells into four clusters based on electrophysiological properties as previously described (Fig. 1*B*; Gale and Murphy 2014). Separation of the fourth and subsequent clusters is no greater than separation of clusters formed from randomly shuffled data (Fig. 1*C*).

**Table 1.** Fraction of sSC cells expressing Cre-dependent reporter in four transgenic mouse lines

	Ntsr1-GN209			Gad2		Rorb	
Cre Line Reporter	AAV GFP	Ai32	Grp-KH288 AAV GFP	AAV GFP	Ai9	AAV GFP	Ai14
WF	16/22	6/6	0/16	0/11	0/6	0/8	0/12
NF	0/9	0/3	10/15	0/8	0/6	0/7	5/10
Horizontal	0/11	0/4	0/13	14/20	11/11	9/16	12/18
Stellate	0/11	0/5	0/13	0/12	0/10	5/11	20/23

Cell types [wide-field (WF), narrow-field (NF), horizontal, and stellate] were identified by their intrinsic electrophysiological properties recorded in brain slices. Various percentages of each cell type expressed Cre-dependent reporter. Reporter expression was not used to target specific cells for recordings. Cre expression was indicated by fluorophore expression in mice crossed to lines with Cre-dependent fluorophore expression (Ai9, Ai14) or injected with virus (AAV) encoding Cre-dependent fluorophore. In Ntsr1-GN209 Cre × Ai32 mice, Cre-dependent ChR2 expression was indicated by sustained spiking in response to blue light (Gale and Murphy 2016). Data from Ntsr1-GN209, Grp-KH288, and Gad2 Cre mice are from Gale and Murphy (2014, 2016). Due to incomplete viral infection in AAV-injected mice, the absence of Cre-dependent reporter expression in any particular cell does not necessarily indicate lack of Cre expression. For example, only ~70% of WF cells (in Ntsr1-GN209 Cre mice) or horizontal cells (in Gad2 Cre mice) were labeled by AAV injections, while 100% of these cells were labeled when the respective Cre lines were crossed to reporter lines. GFP, green fluorescent protein; sSC, superficial layers of the superior colliculus.

In Rorb Cre mice, nearly all stellate cells, approximately half of NF and horizontal cells, and no WF cells were labeled (Table 1). This degree of selectivity, in particular the nearly complete labeling of the stellate cells (20/23 in Rorb Cre x Ai14 mice) and the absence of label in WF cells, combined with previous data is sufficient to allow inferences about the connectivity of stellate cells, as detailed below.

**Fig. 2.**
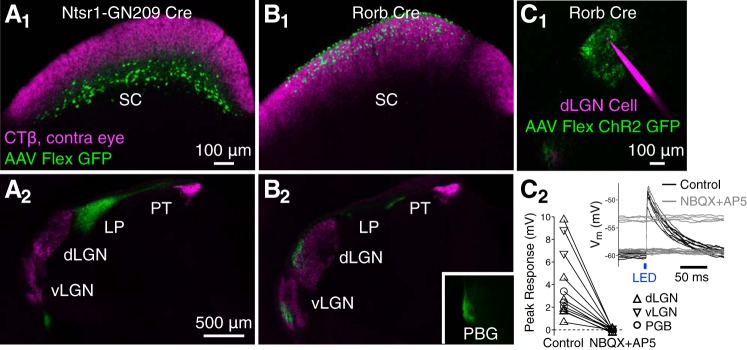
Stellate cells project to lateral geniculate nucleus (LGN) and parabigeminal nucleus (PBG) but not lateral posterior (LP) and are glutamatergic. All images are coronal sections with dorsal up and lateral left. *A*: green fluorescent protein (GFP)-expressing cells (green) at the injection site in the superior colliculus (SC; *A_1_*) and axons in thalamus (*A_2_*) after injection of AAV-Flex-GFP in a Ntsr1-GN209 Cre mouse. Retinal ganglion cell axons (magenta) were labeled via injection of cholera toxin subunit-β (CTβ) conjugated to Alexa 594 in the contralateral eye. GFP-expressing axons below ventral LGN (vLGN) in *A_2_* are following the optic tract in route to the contralateral LP. PT, dorsal pretectum. *B*: same as in* A* for injections in a Rorb Cre mouse. *C*: synaptic responses to blue light (black and gray traces, *C_2_*) in a dorsal LGN (dLGN) cell (magenta, *C_1_*) recorded in vitro following AAV-Flex-ChR2-GFP (green, *C_1_*) injection in the sSC of a Rorb Cre mouse. Example traces were recorded before (black) and after (gray) bath application of the glutamate-receptor antagonists NBQX and AP5. Current injection was used to depolarize the cell for some trials after NBQX/AP5 application. The peak response to ChR2 stimulation, before and after NBQX/AP5 application, is shown in *C_2_* for 7 dLGN (triangles), 2 vLGN (inverted triangles), and 3 PBG neurons (circles).

To compare axonal projections of WF and stellate cells, we injected virus coding for Cre-dependent expression of GFP into the sSC of Ntsr1-GN209 Cre mice, in which WF cells are specifically labeled, or Rorb Cre mice. As previously shown (Gale and Murphy 2014) in Ntsr1-GN209 Cre mice, we observed GFP-labeled axons in ipsilateral and contralateral LP but not dLGN or vLGN (Fig. 2*A*). Unlike our previous results using a different virus preparation, we did not observe retrogradely labeled cells in the PBG. There were no labeled axons in the PBG either, consistent with the observations that *1*) WF cells are not labeled by retrograde tracer injections in the PBG (Gale and Murphy 2014), and *2*) tracer injections in LP and PBG retrogradely label separate groups of sSC cells (Shang et al. 2018). Thus WF cells project to LP (bilaterally) but not to dLGN, vLGN, or PBG.

GFP-labeled axons in Rorb Cre mice labeled a complementary and nonoverlapping set of downstream nuclei: GFP axons were absent in LP but dense in dLGN, vLGN, PBG, and the dorsal pretectum (Fig. 2*B*). Similar labeling is apparent from sSC injections in Rorb Cre mice in the Allen Institute Mouse Brain Connectivity Database (Oh et al. 2014). In dLGN and vLGN, these axons are unlikely to originate from WF cells, which do not express Cre in Rorb Cre mice, or from NF cells, which, when labeled in the Grp-KH288 Cre line, have axons in the PBG but not LGN and are not labeled by retrograde tracer injections in LGN (Gale and Murphy 2014). However, a subset of horizontal cells are labeled in Rorb Cre mice (Fig. 1*B*; Table 1), and some horizontal cells project to dLGN and vLGN. Indeed, axonal projections in Rorb Cre mice appeared similar to those observed in Gad2 Cre mice, which specifically labels horizontal cells (Gale and Murphy, 2014).

To determine whether stellate (glutamatergic) and/or horizontal (GABAergic) cells contribute to the sSC projections in Rorb Cre mice, we injected virus-enabling Cre-dependent ChR2 expression in the sSC of Rorb Cre mice and recorded responses to blue light pulses in brain slices of regions containing labeled axons (Fig. 2*C*). ChR2 stimulation elicited excitatory postsynaptic potentials in all dLGN (*n* = 7), vLGN (*n* = 2), and PBG (*n* = 3) neurons recorded (*n* = 3 mice); these responses were blocked by the glutamate-receptor antagonists NBQX and AP5 (Fig. 2C). In dLGN and vLGN, the probable source of this glutamatergic input is stellate cells, whereas in PBG both stellate and NF cells likely contribute (both are labeled by retrograde tracer injections in PBG; Gale and Murphy, 2014).

Notably, in Rorb Cre mice we did not observe inhibitory postsynaptic potentials (IPSPs) in dLGN, vLGN, or PBG neurons after glutamate receptors were blocked, even when the driving force for GABA-A receptor-mediated conductance was increased via somatic depolarization (Fig. 2*C*). By contrast, when similar experiments are conducted in Gad2 Cre mice, IPSPs were observed in all tested dLGN, vLGN, and PBG neurons; these responses were unaffected by NBQX and AP5 and abolished by the GABA-A receptor antagonist gabazine (Gale and Murphy 2014). Thus horizontal cells that express Cre in Rorb Cre mice are likely interneurons, consistent with the observations that *1*) <5% of GABAergic cells in sSC project to dLGN (Bickford et al. 2015), and *2*) sSC GABAergic cells that receive input from V1 do not project outside of sSC (Zingg et al. 2017).

**Fig. 3.**
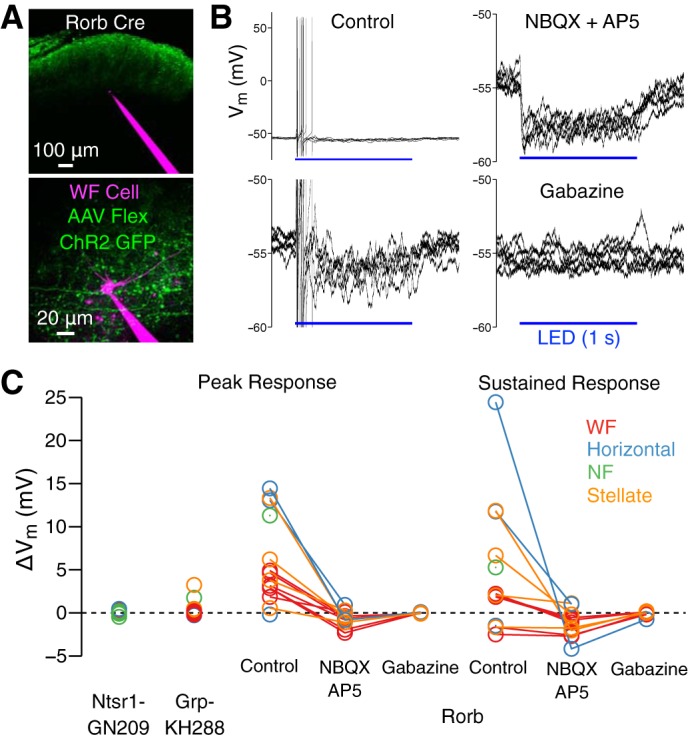
Stellate cells provide excitatory input to other superior colliculus (SC) cell types, including wide-field (WF) cells, and Rorb-Cre horizontal cells make local inhibitory connections. *A*: example WF cell (magenta) recorded in vitro following injection of AAV-Flex-ChR2-green fluorescent protein (GFP) (green) in the SC of a Rorb Cre mouse. *B*: responses of the cell shown in *A* to 1-s pulses of blue light. The *top* and *bottom left traces* show different *y*-axis ranges for trials recorded before drug application. The *top* and *bottom right traces* show trials recorded after bath application of glutamate-receptor antagonists (NBQX and AP5) and then the GABA-A receptor antagonist gabazine, respectively. *C*: peak and sustained responses (see methods) of non-ChR2/GFP-expressing cells recorded in Ntsr1-GN209, Grp-KH288, or Rorb Cre mice. Cell type is indicated by symbol color.

These results demonstrate that sSC projections to LP and dLGN are mediated by distinct cell types. Are these pathways isolated, parallel streams, or do WF and stellate cells interact via local connections within sSC? To answer this question, we injected virus-enabling Cre-depenent ChR2 expression in sSC in each of the four cell-type selective Cre lines (Fig. 1*B*; Table 1) and recorded responses to blue light flashes in non-labeled sSC neurons (Fig. 3). In Gad2 Cre mice, IPSPs were elicited in all cells (*n* = 9 WF, 7 NF, and 10 stellate cells; *n* = 6 mice; Gale and Murphy 2016). In Ntsr1-GN209 Cre mice (labeling WF cells), no responses were observed (*n* = 2 WF, 4 NF, 2 stellate, and 4 horizontal cells; *n* = 4 mice), and in Grp-KH288 Cre mice (labeling NF cells), excitatory postsynaptic potentials were rarely observed (*n* = 0/11 WF, 1/3 NF, 1/6 stellate, and 0/5 horizontal cells; *n* = 9 mice; Fig. 3*C*).

In contrast to experiments in the Ntsr1-GN209 and Grp-KH288 Cre lines, robust synaptic responses were observed in nearly all sSC cells in Rorb Cre mice (*n* = 7/7 WF, 1/1 NF, 4/4 stellate, and 3/4 horizontal cells; *n* = 5 mice). Brief blue light pulses (<10 ms) elicited a depolarizing response (Fig. 3*C*, “peak response”). Similarly, longer blue light pulses (1 s) elicited a transient depolarization (often accompanied by action potentials) followed by sustained depolarization or hyperpolarization (Fig. 3, *B* and *C*, “sustained response”). The depolarization was blocked by NBQX and AP5 (*n* = 12), often revealing a stronger sustained hyperpolarization that was blocked by gabazine (*n* = 7; Fig. 3, *B* and *C*). Which sSC cell types contribute to these responses? GABAergic responses are clearly attributable to the only GABAergic cell type, horizontal cells. Given relatively rare and weak synaptic responses in Grp-KH288 Cre mice (labeling NF cells), stellate cells are the probable source of glutamatergic responses in Rorb Cre mice. Thus, although different cell types give rise to sSC projections to LP and dLGN, there is an excitatory interaction between these pathways via a stellate-to-WF cell connection.

## DISCUSSION

**Fig. 4.**
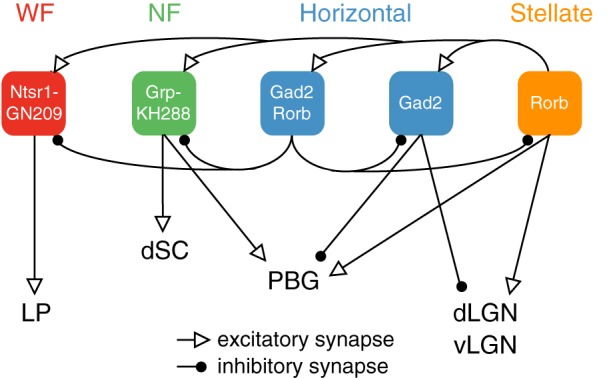
Summary schematic of superficial layers of the superior colliculus (sSC) connectivity. Cell types are indicated by color. Transgenic lines in which each cell type expresses Cre are indicated by white text in colored shapes. Lines represent excitatory (arrowhead) and inhibitory (circles) connections. Dorsal and ventral lateral geniculate nuclei (dLGN and vLGN) are combined because we did not find differences in sSC connections to these regions. LP, lateral posterior; dSC, deeper superior colliculus; WF, wide-field cells; NF, narrow-field cells.

Our current working model of sSC connectivity, bolstered by results described in this paper, is summarized in Fig. 4. One of the most important points relevant to understanding the functions of sSC is that two distinct cell types give rise to excitatory projections to LP (WF cells) and dLGN (stellate cells). The projection from WF cells to LP is specialized in the sense that WF cells do not project to any other targets and no other sSC cell types project prominently to LP. In contrast, stellate cells provide input to other sSC neurons and multiple targets outside of sSC and share each output with at least one other sSC cell type. Individual stellate cells, however, might form connections with specific subsets of the cells/regions targeted by the entire population of stellate cells (Diamond et al. 1991).

It is important to note that we cannot completely rule out the possibility that a small subset of NF cells project to LP or LGN. As noted previously, the axons of NF cells labeled in Grp-KH288 Cre mice follow the optic tract dorsal of LP but do not ramify ventrally into LP or continue laterally to LGN (Gale and Murphy 2014). This could reflect a sparse but functionally relevant projection to the dorsal-most part of LP. While the Grp-KH288 Cre line might not label all NF cells, the fact that we did not observe retrogradely labeled NF cells following tracer injections in LP or dLGN suggests that a small fraction of NF cells, if any, project to these targets.

Most if not all neurons in posterior LP receive glutamatergic input from WF cells (Gale and Murphy 2014; Masterson et al. 2010). Moreover, multiple WF cell axons converge on single neurons with clusters of large terminals forming synapses on proximal dendrites (Bickford 2016; Masterson et al. 2009; Zhou et al. 2017). Hence, WF cells may play a major role in determining the output of posterior LP to visual cortex. The relative impact of WF cell and cortical input to LP, however, has not been examined directly.

Stellate cells provide strong synaptic input to the dorsolateral shell region of dLGN (Bickford et al. 2015). Interestingly, direction-selective retinal ganglion cells target both the shell region of dLGN and the superficial-most layer of sSC (Cruz-Martín et al. 2014; Kay et al. 2011; Rivlin-Etzion et al. 2011), which is enriched in both stellate cells and direction-selective neurons (Inayat et al. 2015). Stellate cells might contribute to circuits carrying information about direction of motion to primary visual cortex.

Although distinct cell types mediate sSC output to LP and dLGN, stellate-to-WF cell connections allow for possible functional interaction between these pathways. In contrast to the robust connection of stellate cells to other sSC neurons, however, intra-sSC connections originating from other excitatory sSC cell types (NF and WF cells) were rare or absent in our data set. It is possible that such connections exist but are less common, weak, and/or form synapses on distal dendritic processes (which might preclude detection via somatic recordings). Data from parvalbumin Cre mice, which like Rorb Cre mice appear to label multiple sSC cell types including stellate cells, also demonstrate excitatory interactions between sSC neurons (Shang et al. 2018; Villalobos et al. 2018).

NF cells labeled by the Grp-KH288 Cre line project a dense band of axons to deeper SC layers (dSC; Gale and Murphy 2014) that is not observed in Ntsr1-GN209 or Rorb Cre mice. Nonetheless, we cannot rule out the possibility that WF, stellate, or horizontal cells also link sSC to dSC. Connections between excitatory neurons expressing ChR2 in Rorb Cre mice and dSC neurons, if they exist, could contribute to the inhibitory component of the synaptic response we recorded in sSC (Fig. 3). In this scenario, ChR2 stimulation of sSC Rorb Cre neurons would directly activate excitatory and inhibitory sSC neurons as well as indirectly excite dSC inhibitory neurons that project back to sSC (Lee et al. 2007; Phongphanphanee et al. 2011). Excitatory connections from dSC to sSC are uncommon and target a small population of non-WF cells in the deeper part of the sSC (Ghitani et al. 2014); it is unlikely these connections contributed to the synaptic responses we recorded in sSC.

It is likely that future studies will reveal additional diversity of sSC cell types and connectivity. In particular, cell types and connectivity may vary across: *1*) different depths within the sSC, which receive input from distinct subsets of retinal ganglion cell types (Kim et al. 2010), and/or *2*) positions representing different portions of visual space, which, in the dSC at least, differ anatomically and might have distinct functional roles (Comoli et al. 2012; Dean et al. 1989).

The dSC is commonly associated with a role in targeting and initiating eye movements, but more recent experiments reveal much broader functions (Crapse et al. 2018; Lovejoy and Krauzlis 2010; Zénon and Krauzlis 2012). The functions of the sSC are unknown, but it receives input from nearly every retinal ganglion cell and visual cortical area and sends outputs to major visual thalamo-cortical circuits. Elucidating the functional impact of sSC on these circuits may reveal important insights into how brains use visual information to guide behavior.

## GRANTS

This work was supported by the Howard Hughes Medical Institute.

## DISCLOSURES

No conflicts of interest, financial or otherwise, are declared by the authors.

## AUTHOR CONTRIBUTIONS

S.D.G. and G.J.M. conceived and designed research; S.D.G. performed experiments; S.D.G. analyzed data; S.D.G. and G.J.M. interpreted results of experiments; S.D.G. prepared figures; S.D.G. drafted manuscript; S.D.G. and G.J.M. edited and revised manuscript; S.D.G. and G.J.M. approved final version of manuscript.
